# Rationale for Increasing Oncological Vigilance in Relation to Clinical Findings in Accessory Parotid Gland—Observations Based on 2192 Cases of the Polish Salivary Network Database

**DOI:** 10.3390/cancers16020463

**Published:** 2024-01-22

**Authors:** Małgorzata Wierzbicka, Ewelina Bartkowiak, Wioleta Pietruszewska, Dominik Stodulski, Jarosław Markowski, Paweł Burduk, Izabela Olejniczak, Aleksandra Piernicka-Dybich, Małgorzata Wierzchowska, Katarzyna Amernik, Alicja Chańko, Daniel Majszyk, Antoni Bruzgielewicz, Patrycja Gazinska, Bogusław Mikaszewski

**Affiliations:** 1 Department of Otolaryngology, Regional Specialist Hospital Wroclaw, Research & Development Centre, 51-124 Wroclaw, Poland; wierzbicka.otolaryngology@gmail.com; 2 Faculty of Medicine, Wroclaw University of Science and Technology, 50-370 Wroclaw, Poland; 3 Institute of Human Genetics, Polish Academy of Sciences, 01-447 Poznan, Poland; 4 Department of Otolaryngology and Laryngological Oncology, Poznan University of Medical Sciences, 61-701 Poznan, Poland; ewelina.anna.bartkowiak@gmail.com; 5 Department of Otolaryngology, Head Neck Oncology, Medical University of Lodz, 90-419 Lodz, Poland; izabela.olejniczak@umed.lodz.pl; 6 Department of Otolaryngology, Faculty of Medicine, Medical University of Gdansk, 80-210 Gdansk, Poland; dstodulski@gumed.edu.pl (D.S.); boguslaw.mikaszewski@gumed.edu.pl (B.M.); 7 Department of Laryngology, Faculty of Medical Sciences in Katowice, Medical University of Silesia, 40-055 Katowice, Poland; jmarkow1@poczta.onet.pl (J.M.); laryngologia@spskm.katowice.pl (A.P.-D.); 8 Department of Otolaryngology, Phoniatrics and Audiology, Collegium Medicum, Nicolaus Copernicus University, 87-100 Bydgoszcz, Poland; pburduk@cm.umk.pl (P.B.); wierzchowskam@op.pl (M.W.); 9 Department of Otolaryngology, Pomeranian University of Medicine, 70-204 Szczecin, Poland; jaworowska@tlen.pl (K.A.); qba555p@gmail.com (A.C.); 10 Department of Otorhinolaryngology Head and Neck Surgery, Medical University of Warsaw, 02-091 Warsaw, Poland; majszykdaniel@gmail.com (D.M.); abruzgielewicz@wum.edu.pl (A.B.); 11 Biobank Research Group, Lukasiewicz Research Network—PORT Polish Center for Technology Development, Stabłowicka St., 147, 54-066 Wroclaw, Poland; patrycja.gazinska@port.lukasiewicz.gov.pl

**Keywords:** accessory parotid gland, APG, region V, salivary gland, parotid gland tumours

## Abstract

**Simple Summary:**

The accessory parotid gland differs in histological structure from main parotid tissue, making the appearance of tumours different than those in the rest of the gland. Therefore, our aim was to analyse the epidemiological and histological differences of parotid tumours located in regions I–V, with particular emphasis on the distinctiveness of region V. Furthermore, to define the epidemiological factors that will indicate the risk of histological malignancy from clinically benign appearances was our object of interest. We confirmed our hypothesis that the epidemiology, clinical behaviour, histology, tactics of the surgical procedure, route of access, and prognosis differ in the parotid gland tumours located in superficial (region I, II) and deep (region III, IV) lobes, as well as the accessory lobe (region V), despite originating from the one organ.

**Abstract:**

The accessory parotid gland (APG, Vth level) differs in histological structure from main parotid tissue. This gives rise to the hypothesis, mirrored in clinical observations, that the representation of tumours is different than in the rest of the gland. The aim of the study was to analyse the epidemiological and histological differences of parotid tumours located in regions I–V, with particular emphasis on the distinctiveness of region V. To define the epidemiological factors that will indicate the risk of histological malignancy from clinically benign appearance, multicentre prospective studies conducted between 2017–2021 by five Head and Neck Surgery University Departments, cooperating within the Polish Salivary Network Database 1929 patients (1048 women and 881 men), were included. The age, gender, patient occupation, place of inhabitation, tumour size, clinical features of malignancy, histology, and facial nerve (FN) paresis were analysed for superficial (I_II) and deep (III_IV) lobes and with special regard to the tumours affecting region V. Twenty eight tumours were located exclusively in region V (1.45% total) and seventy-two tumours were found in region V exhibiting extensions to neighbouring regions (3.7% total), characterised as significantly younger and less frequent in retirees. In I–IV regions, approximately 90% of tumours were benign, with pleomorphic adenoma (PA) and Whartin tumour (WT) predominance. In region V, PA exceeded 75% but WT were casuistic (2/28). Incidences of malignancies in region V was 40% but clinical signs of malignancy were evident only in tumours > 4 cm or in the presence of FN paresis. In 19% of patients with a benign appearance, imaging revealed malignancy; however, 38% of patients showed false negative results both in terms of clinical and radiological features of malignancy. Logistic regression models in 28 patients with tumours located exclusively in region V vs. 1901 other patients and in 100 patients with V extension vs. 1829 other patients showed no clinical symptoms of malignancy binding with final malignant tumour histology as a single variable or in combination with other variables. The logistic regression models obtained in this study show strong linkage between tumour location and predictors (age, male gender, and tumour diameter) and also aimed to function as a good classifier. Our conclusion is that, despite the very clear image of the mid-cheek tumour which is easily accessible in palpation and ultrasound examination, it is necessary to improve oncological vigilance and preoperative patient preparation.

## 1. Introduction

The accessory parotid gland (APG), a salivary tissue separated from the main gland and lying on the masseter muscle, exists in 21–61% of the population [1,2]. It has a secondary duct which empties into the Stensen’s duct. The clinical findings of parotid anterior extension were confirmed recently in a systematic computed tomography scan analysis [3]. The distinctiveness of the APG is reflected in the classification established by the Third Meeting of the European Salivary Gland Society (ESGS). The ESGS proposes to subdivide the parotid parenchyma into five levels: I (lateral superior), II (lateral inferior), III (deep inferior), IV (deep superior), and V (accessory) [4]; this proposal has been followed by others [5].

Determinants of the anatomical and histological structure of the parotid take place in embryogenesis [6,7]. The pattern of differentiation of a significant fraction of APG differs from that of the main parotid. It appears that mixed acini, present in the early stages of development, persist into later life, and their presence may be related to tumours developing at these sites [2]. This gives rise to the hypothesis, mirrored in clinical observations, that the representation of tumours in region V is different than in the rest of the gland.

A typical region V tumour appears as a non-tender, slowly growing, mid-cheek mass, with a median diameter of 1 cm, developing exceptionally for a couple of months with rapid increases in size [8,9]. In the 1990s, the behaviour of these tumours and the spectrum of neoplasia was thought to be similar to those arising from the main gland [10]. New knowledge in the world literature over the past decades has indicated some differences, but experiences were scarce and mainly based on case reports [8,11,12] or sets of cases [13,14,15,16,17,18]. The largest single centre cohort analysed 130 APG tumours, of which 23.8% were malignant, doubling the established classic tumour rates for the main gland [19]. An even higher percentage of malignancies was presented in a recent meta-analysis, where region V benign tumours made up 61.5% of cases [20].

The first choice of treatment for APG tumours is surgical resection. The specificity of the location of the accessory lobe around Stensen’s duct and the immediate vicinity of the masseter muscle implies a need for an alternative surgical approach. Access to region V is difficult and the procedure may carry the risk of damaging the VII buccal branch as well as Stensen’s duct. Methods of accessing region V have evolved over the decades. In the early 1990s, the cheek incision was promoted on the grounds of a location that was difficult to achieve for the classic Blair incision. The cheek incision versus face lift incision was considered equally good [10]. From the turn of the century, a standard face lift approach, a modified Blair incision [15], or a standard parotidectomy incision with anterior extension [17] has been used. A transoral approach is known to mitigate patients’ cosmetic concerns [21,22]; however, prior reports utilised endoscopic assistance on patients with smaller tumours [23]. The endoscopic-assisted technique has proven useful to achieve tumour resection while avoiding serious complications due to surgical procedures [24,25]. Nevertheless, a current standard parotidectomy approach is still thought to be safe and cosmetically appealing [14,15,17].

Hypothesis: The epidemiology, clinical behaviour, histology, tactics of the surgical procedure, route of access, and prognosis differ in the parotid gland tumours located in superficial (region I, II) and deep (region III, IV) lobes, as well as the accessory lobe (region V), despite originating from the one organ.

Objective: To analyse the epidemiological and histological differences of parotid tumours located in regions I to V, with particular emphasis on the distinctiveness of region V. Also, to define the epidemiological factors that will indicate the risk of histological malignancy from clinically benign appearances.

## 2. Materials and Methods

A multicentre prospective study was conducted over a 5-year period (2017–2021) by five Head and Neck Surgery University Departments, cooperating within the Polish Salivary Network Database. Overall, 2192 patients with large salivary gland tumours, including 1205 women and 987 men, were analysed. For 263 patients, there was no clear indication of the region from which the tumour had been removed and were excluded from this analysis. Finally, 1048 women and 881 men were included in the analysis, giving a total of 1929 patients for a detailed description of parotid tumours.

To achieve the main goal of this research, differences between tumour location in region V, i.e., APG, versus other locations were analysed. Thus, the first step was to categorise the patients in terms of tumour location: limited to region V, affecting region V and the neighbouring regions, and with no extension to region V.

The age, gender, patient occupation, place of inhabitation, tumour size, clinical features of malignancy, histology, radical surgery, and facial nerve (FN) paresis were analysed for superficial lobes (I_II), deep lobes (III_IV), and with special regard to the tumours exclusively affecting region V and affecting both region V and the neighbouring regions. In our research, a feature that clearly and unambiguously indicates malignancy, such as nerve palsy, was cited mainly as an indicator of malignancy and a symptom prompting medical attention. Since a detailed analysis of symptoms characteristic of malignant tumours, their severity, and their duration was not the subject of this publication, the logistic regression analysis presented below concerned only one feature most typical of malignant tumours, i.e., FN paresis/paralysis. Tumours were considered benign if they did not have the above feature of malignancy. We analysed the tumour size assessed from macroscopy once surgical removal had been performed. To analyse the relationships between variables, the chi-square test of independence, G test, and Fisher’s exact test were used. The Yates and Williams correction methods were used regarding chi-square and G tests, respectively. In order to perform post hoc tests with multiple comparisons, the chi-square test of independence with Holm correction and Fisher’s exact test considering *p* values < 0.05 statistically significant were used.

In order to establish relationships between response and predictor variables, the response variable was related to the location of the tumour and its anatomical configuration as defined in the cohort of studied patients, which are as follows: Group A, patients with a tumour located exclusively in region V versus the rest of the patients; Group B, patients with a tumour located in region V exclusively and with extension to neighbouring regions versus the rest of the patients. The predictor variables used in the analysis were age, sex, tumour size, histology, and clinical characteristics of malignancy. First, two separate models consisting of all predictor variables were created and applied to both predictor response variables (Group A and B). Then, reduced models with selected predictor variables were built. For the full models, the significance threshold of *p* = 0.2, while for the reduced model, *p* = 0.05 was adopted. If the statistical significance level was below the adopted thresholds for particular response variables, the hypothesis of non-significance of this factor was rejected. In other words, this variable has a statistically significant impact on the response. In our study, it is the presence of tumours in region V exclusively and in neighbouring regions. Moreover, if more than one predictor variable which is statistically significant influences the response in a particular model, there is also a relationship between such dependent variables.

## 3. Results

From a total of 1929 parotid tumours, 28 tumours were located exclusively in region V and an additional 72 tumours were found in region V with extension to neighbouring regions (Table 1).

Analysis of all combinations of tumour locations and extension registered in the database showed the predominance of superficial lobes: II—(*n* = 663), I—(*n* = 85), I_II—(*n* = 473). The second most frequent location was in the lower pole of the parotid, covering regions II_III—(*n* = 224) and III—(*n* = 58). Scattered patient numbers were obtained for having the most extensive tumours in multiple regions simultaneously. The variables concerning patients’ demographic, tumours, surgery, and histology are presented in Table 2.

### 3.1. Age

Age analysis for the whole group showed an average of 59.6 years. The details of the mean and median age of patients with tumours in the superficial (I, II) and deep (III, IV) regions as well as region V are shown in Table 2.

After categorising the patients in terms of tumour limited to the V region, and both subgroups together; limited to region V and V + other regions, (over mean age, 44 (44.4%); below mean age, 55 (55.55%)) versus other locations (over mean age, 1069 (58.44%); and below mean age, 760 (41.55%)), statistically significant differences were found (Fisher Test *p* = 0.006). In patients with tumours limited to region V, there were 10 (35.71%) over mean age and 18 (64.28%) below mean age; the difference was statistically significant (Fisher Test *p* = 0.0019).

The most prevalent occurrence of tumours in region V for a younger age was confirmed by the analysis of the age median. For the whole group, the median age was 63.4 years. For tumour locations in region V (over median age 36 (36.36%), below 63 (63.63%)) versus other locations (below median 901 (49.26%), above median 928 (50.73%)), statistically significant differences were found (Fisher Test *p* = 0.007). There were no differences found when comparing the median age between other regions: I_II vs. I_II_III (*p* > 0.05), I_II vs. II_III (*p* > 0.05), I_II_III vs. II_III (*p* > 0.05).

### 3.2. Gender

After categorising the patients in terms of tumour limited to region V (61 (61%) female, 39 (39%) male) versus other locations (987 (53.96%) female, 842 (46.03%) male), no statistically significant differences were found between the genders (Fisher’ Test *p* > 0.05). Regarding sex, even if the differences between males and females are not statistically significant, given the small number of patients with tumours in the V region, there is a notable difference between the latter and the rest of the patients with a fair prevalence of females in patients with tumours in the V region (61% vs. 53.9%); this requires further observation.

### 3.3. Occupation

The professional activity of patients with salivary gland tumours was analysed, categorising them into white collar workers, manual workers, and retirees. Students were included as white-collar workers.

After categorizing the patients in terms of tumour location (region V versus other locations), there were 35 (40.22%) white collar workers, 21 (24.13%) manual workers, and 31 (35.63%) retirees with V extension tumours. There were 404 (28.03%) white collar workers, 388 (26.29%) manual workers, and 649 (45.03%) retirees with no V extension. The differences were significant (Fisher Test *p* = 0.056; chi-square test *p* < 0.05).

### 3.4. Place of Residence

Place of residence was analysed using four categories: city 100,000–500,000, city > 100,000, city < 500,000, village. They were, respectively, inhabited by 359 (23.11%), 565 (36.38%), 214 (13.77%), and 415 (26.72%) patients. For patients with tumours located in region V, there were no significant differences in residential locations (Fisher Test *p* > 0.05).

### 3.5. Tumour Size and Postoperative Facial Nerve Pulsy

The relationship between tumour size and location was investigated. The tumour size was categorised as <2 cm, 2–4 cm, and >4 cm. There were 32 (32.65%), 55 (56.12%), and 11 (11.22%) tumours affecting the V region in the listed categories. There were 486 (27.1%), 1015 (56.6%), and 292 (16.28%) tumours with no extension to region V in the listed categories. Tumours affecting region V and neighbouring regions with a diameter > 4 cm were the most numerous, but the differences between tumour size and location did not reach significance (Fisher Test *p* > 0.05).

Considering tumours limited to region V, the prevalence differs significantly (Fisher Test *p* = 0.006). There were 13 (48.14%), 14 (51.85%), and 0 patients in the listed size categories. No patients had tumours larger than 4 cm.

Postoperative facial nerve pulsy was observed only in two patients with tumours affecting the V region and neighbouring regions (Table 3).

### 3.6. Histopathology

The results of histological examinations are presented in Table 4 and Table 5. The histological specimens assessed included 1658 benign and 116 malignant tumours of salivary gland origin. The rest of the lesions were extraparotid tumours located in the preauricular region, for example, lipoma, lymphangioma, or atheroma.

Benign tumours were categorised as pleomorphic adenoma (PA), Whartin tumour (WT), and other histological diagnoses. There were 47 benign and 16 malignant tumours affecting region V. Regarding tumours with no extension to region V, there were 1588 (94.07%) benign and 100 (5.92%) malignant tumours. The difference was statistically significant (Fisher Test 7.964352 × 10^−5^ *p* < 0.0001).

There were five malignant and twenty-three benign tumours limited to V region. Regarding the tumours with no extension to region V, the difference was statistically significant (Fisher Test *p* = 0.024).

There were 802 (48.63%) PA, 741 (44.93%) WT, and 106 (6.43%) of other histology with no extension to the Vth region. There were 60 (72.29%) PA, 9 (10.84%) WT, and 14 (16.87%) of other histology affecting the V region. The differences were statistically significant (Fisher Test 1.342137 × 10^−9^ *p* < 0.0001). Fisher’s Test and chi-square with Holm’s correction for multiple variables were used to compare PA vs. WT and other types vs. WT. These showed significant statistical differences in the higher incidence of PA compared to WT and a higher percentage of other histology versus WT for region V.

### 3.7. Satellite Foci

Satellite foci were found in ninety-seven patients’ postoperative specimens, in ninety-one (6.13%) without, and in six (8.45%) with extensions to region V. No satellites were found in 1393 (93.86%) without and in 65 (91.54%) with extensions to region V. Although there was a higher percentage of satellites for region V extension, the difference was not significant (Fisher Test *p* > 0.05). Tumours limited to region V presented no satellite foci versus 21 localised in regions I_IV, but the score was not significant.

### 3.8. Surgery Technique and Surgical Margins

A standard parotidectomy (Blair incision) with anterior extension was applied in all 100 V region tumours. None of the departments used the intraoral approach.

The relationship between safe margins and region V location was investigated. Tumour margins were categorised as R0, R1, R2, and there were seventy-one (87.65%), six (7.4%), and four (4.93%) cases in the listed categories, respectively. There were 1408 (91.3%), 82 (5.31%), and 52 (3.37%) tumours with no extension to region V in the listed categories. There was no statistically significant relationship between examined tumour locations and safe margins (Fisher Test *p* = 0.447). Tumours limited to region V were R0 in twenty and R1 in two cases, with no occurrence of R2 patients.

The logistic regression models were used in order to establish relationships between predictor variables and response were presented. The predictor variables used in the analysis were: age, gender, size of the tumour, histology and clinical features of malignancy and were all included to create two full models. The response variables are as follows: Group A patients with tumours located exclusively in region V versus the rest of the patients (Table 6), and Group B patients with tumours located in region V exclusively and with extension to neighbouring regions versus the rest of the patient (Table 6 and Table 7).

Then, we built reduced models with selected response variables.

All variables can potentially depend on each other. In Group A, coupled variables of male gender and age, male gender and malignant histology, malignant histology and tumour diameter > 2 cm affect region V location, but the other combinations did not. In Group B, coupled variables are age, tumour diameter > 2 cm, and malignant histology. In a model that includes age and gender, the two variables interact such that male gender and lower age are associated with location in region V. Similarly, age and malignant histology and tumour diameter > 2 cm interact and show strong linkage between selected response variables and predictor (Table 7). Although there is an association with tumour 2–4 cm (*p* < 0.05) assessed macroscopically after surgery, this size is not a risk factors for type V.

Both logistic regression models, the first one in 28 patients with tumours located exclusively in region V vs. 1901 other patients and the second one, in 100 patients with V extension vs. 1829 other patients, showed no clinical symptoms of malignancy binding with final malignant tumour histology as a single variable or in combination with other variables.

The model obtained in this study shows linkage between tumour location and predictors (age, male gender, and tumour diameter) and aimed to function as a good classifier.

## 4. Discussion

We presented a unique analysis of a cohort of 1929 parotid gland tumours, with a more detailed focus on 100 tumours confined to the accessory parotid gland. In our studies, we took a novel approach of distinguishing and analysing region V, which represents some distinctive clinical features and, as yet, has no reference in the literature. We included tumours solely in region V, and tumours spanning multiple regions including region V (tumours with V extension). The latter entity included various combinations of regions I–IV and V region, accounting for over 3.7% total of all patients. Extensive tumours predominated in this group; more than 50% of them were >4 cm in diameter, and it was difficult to determine whether they originated in region V or rather penetrated during the growth of the tumours from other regions of the parotid. Hamza and Anand (2023) recommend the choice of surgical access depending on the location of the tumour in the mid-cheek [26].

In our cohort, tumours confined to region V were rare, accounting for a total of 1.45%. Our results confirm earlier ones, where percentages of APG tumours range from 1 to 7.7% [3,27,28,29].

We were able, in our studies, to document new findings, such as tumours in region V occurring at a significantly younger age than in other regions of the parotid. This was confirmed by patients’ occupation analysis, as there were no differences between white-collar workers and manual workers, but region V tumours were significantly less frequent in the group of retirees. This finding is difficult to compare with other studies because, so far, no emphasis has been placed on the age of tumour development in relation to the location in the parotid.

Surgical access to region V tumours and the complication rate of FN palsy remains a matter of debate. APG resection with concurrent partial parotidectomy and resections limited to the APG had a low overall complication rate of 6.3% and 8.7%, respectively [20]. In our study, the rate of postoperative FN palsy was comparable between tumours with V extension, which represents the majority of cases, and other locations. Of note was the absence of FN palsy in tumours confined to region V.

Alongside the functional findings, final histology data obtained from the surgical specimens are of importance. Routinely assessed resection margins and the presence of satellite foci are prognostic factors and risk factors for recurrence. The percentages of the surgical margins, categorised as R1, R2, and the presence of the satellite foci in postoperative specimens were higher in region V compared to tumours with no extension to region V, although the scores did not achieve statistical significance.

Masses in region V have complex histopathological types, which derive from the cellular diversity in the salivary gland, but also the result of a distinct type of differentiation of the APG parenchyma from the rest of the gland [2,30,31]. From this point of view, it is of importance to compare the characteristics of tumours limited to region V versus I–IV regions, i.e., with all other locations in the salivary gland parenchyma. To take a closer look at the specificity of superficial, deep, and APG tumours, those limited to region V versus I–II and III–IV were compared. In superficial, deep, and combinations of other locations excluding region V, approximately 90% of lesions were benign. The most common histological type was pleomorphic adenoma (PA) and Whartin tumour (WT), similar to other described cohorts [Ma]. PA accounted for over half of the benign tumours in superficial regions, exceeding 60% in the deep lobe and in region V, and there was a strong predominance of tumours spanning region V, exceeding 75% of histological findings. WT were casuistic (2/28) in the isolated APG location.

The analysis of the malignant nature of the tumours limited to region V account for nearly 18% and is similar to the results reported by other authors [19,27,28], although some authors report even higher rates of malignant tumours [13,32]. Noteworthy is the high incidence of malignancies in the subgroup of tumours with V extension, coming in at 40%. The distribution of histology results is striking, with typical salivary gland malignancy predominating; MALTomas are also present. The above-described tendency for large tumour sizes in this subgroup, together with the combination of a high percentage of malignancies and a relatively younger age of patients, may indicate a combination of high-risk factors and requires increased oncological vigilance.

Another key factor for the correct diagnosis of neoplastic processes is a detailed analysis of discrepancies between clinical features and the final histological picture. Clinical signs of malignancy were evident only in tumours larger than 4 cm at diagnosis or in the presence of facial nerve paresis. In 19% of patients with a benign clinical picture, advanced imaging studies revealed malignancy; however, in 38% of patients, the result remained false negative both in terms of clinical and radiological features of malignancy. Fine-needle biopsy (FNB) should be remembered and underlined as a less invasive option. Although many studies have shown that FNB often fails to disclose the histological type of the tumour in advance, it almost always reveals its possible malignancy [33]. This finding may possibly lead to some changes in the clinical protocols, more invasive diagnostic approach, and quicker interventions.

Finally, we proposed logistic regression models to establish relationships between predictor variables and responses. The model coupled variables were male gender and age, male gender and malignant histology, malignant histology, and tumour diameter > 2 cm with the response variable (tumour location in region V versus the rest of the patient). We found that more than one predictor variable significantly statistically influences the predictor in our model: younger age, male gender, and tumour diameter > 2 cm. This indicates that the obtained model is a good classifier and shows strong linkage between selected predictor variables and response, i.e., tumour location.

But even more important is the variable that did not reach statistical significance in the presented models: “no clinical symptoms of malignancy”. Both logistic regression models showed that the variable “no clinical symptoms of malignancy” has not been bound with final malignant tumour histology as a single variable or in combination with other variables. This finding suggests that final histology cannot be inferred from the clinical features of the absence of malignancy and the benign macroscopic morphology of the tumour.

Thus, even more important is the combination of epidemiological premises included in the presented classifier to predict/make a suspicion of malignancy in the Vth region tumours.

To summarise, on the one hand, the value of our research consists of confirming facts that are known and seemingly obvious to surgeons with extensive clinical practice on a large group of patients. At this point, a significantly higher incidence of malignancies in region V should be mentioned. Nevertheless, these observations in the literature have so far been based on smaller patient series, whereas our study had the advantage of a multicentre systematic analysis of a large group of patients. Further value from our innovative observations were the basis for determining the algorithm for unfavourable epidemiological features of tumours of region V. Tumours limited to region V are characterised by a younger age of patients, higher percentage of malignancies, and a high number of clinically false negative cases characterised by a mild clinical picture not followed by radiological proof, but malignant in pathology. The negative prognostic factors, i.e., higher percentage of positive margins and satellite foci, make this group of apparently easy to visualise tumours insidious and difficult. Those tumours with region V extensions also affect younger patients, where much larger size tumours resemble the phenomenon of an iceberg accompanied by other negative features, like high rates of malignancy and unfavourable prognostic factors in histological examination.

However, our work has some limitations, as we were only able to analyse variables that were available and fully documented by all Polish Salivary Network Database members [34,35,36]. This meant that variables such as imaging outcomes could not be included because each department had different equipment, producing separate imaging protocols. Similar difficulties arose in the analysis of fine-needle biopsies, for which the data were fragmentary, and we had to omit their analysis in this study. In addition, we had to exclude several patients due to incomplete data on the analysed variables. Future multicentre studies with more prevalent AI technologies will look towards standardization [37]; future data digitalization should mitigate these kinds of problems.

## 5. Conclusions

As shown above, specifically with regard to variables such as younger age and uncertain biology, it can be concluded that, despite the very clear image of the mid-cheek tumour which is easily accessible in palpation and ultrasound examination, it is necessary to improve the oncological vigilance and preoperative patient preparation.

## Figures and Tables

**Table 1:** Number of tumours limited to the Vth region and affecting the V region and the V + other neighbouring regions.

Localisation/Extension	No of Pts/Percentage *
V (limited to the Vth)	28 (1.45%)
V + other (spanning to neighbouring regions)	72 (3.74%)
I_V	17 (0.88%)
I_II_V	19 (0.98%)
I_II_III_IV_V	10 (0.52%)
I_II_III_V	2 (0.1%)
I_II_IV_V	2 (0.1%)
I_III_IV_V	1 (0.05%)
I_IV_V	1 (0.05%)
II_III_V	3 (0.15%)
II_V	10 (0.52%)
III_V	2 (0.1%)
IV_V	1 (0.05%)
III_IV_V	4 (0.20%)

* % refers to the whole group of 1929 patients.

**Table 2:** Summary of the individual epidemiological variables analysis in patients with different parotid tumour locations.

Tumour Location in I–V Parotid Gland Region	I_II	III_IV	II_III	Limited to V Region	V + Other Regions	Other Location
No. of patients	473	34	224	28	72	1098
Age						
Mean	59.9	58.5	61.0	53.2	56.1	59.9
Median	63.6	63.1	64.3	50.7	56.6	63.5
Occupation						
Collar workers	119	16	38	11	24	231
Manual workers	110	4	39	4	17	235
Retirees	171	11	76	7	24	391
* Incomplete data	73	2	71	6	7	241
Place of residence						
100–500 tys	116	4	24	8	11	215
>100 tys	136	11	75	10	23	343
<500 tys	56	8	27	4	12	123
village	112	9	43	3	20	251
* Incomplete data	53	2	55	3	6	166
Tumour size						
<2 cm	126	9	42	13	19	309
2–4 cm	259	21	136	14	41	599
>4 cm	69	2	43	0	11	178
* Incomplete data	19	2	3	1	1	12

* Number of patients for whom specified data were missing.

**Table 3:** Postoperative facial nerve palsy.

Tumour Location in I–V Parotid Gland Region	I_II	III_IV	II_III	Limited to V Region	V + Other Regions	Other Locations
Yes	12 (2.83%)	1 (3.7%)	5 (3.14%)	0 (0%)	2 (2%)	27 (2.82%)
No	411 (97.16%)	26 (96.29%)	174 (96.85%)	22 (100%)	48 (96%)	930 (97.17%)
*	50	7	45	6	22	141

* number of patients excluded from analysis for whom specified data were missing.

**Table 4:** Summary table for the analysis of histology data of patients with different parotid tumour locations. * number of patients excluded from analysis for whom specified data were missing.

Tumour Location in I–V Parotid Gland Region	I_II	III_IV	II_III	Limited to V Region	V + Other Regions	Other Locations (Combination I–IV)
Tumour histology						
Malignant	19 (4.2%)	1 (3%)	13 (6.37%)	5 (17.85%)	16 (33.95%)	67 (6.7%)
Benign	433 (95.8%)	32 (97%)	191 (93.62%)	23 (82.14%)	47 (77.04%)	932 (93.3%)
*	32	1	20	0	9	99
WT	180 (40.81%)	11 (33%)	97 (48.5%)	2 (7.14%)	7 (12.72%)	453 (46.46%)
PA	236 (53.51%)	20 (60%)	89 (44.5%)	19 (67.85%)	41 (75.54%)	457 (46.87%)
other	25 (5.66%)	2 (6%)	14 (7%)	7 (25%)	7 (12.72%)	65 (6.66%)
*	32	1	24	0	17	123
Margins						
R0	341 (71.48%)	25 (100%)	181 (93.29%)	20 (90.9%)	51 (86.44%)	861 (91.98%)
R1	35 (7.33%)	0 (0%)	7 (3.6%)	2 (9.1%)	4 (6.77%)	40 (4.27%)
R2	11 (2.3%)	0 (0%)	6 (3.09%)	0 (0%)	4 (6.77%)	35 (3.73%)
*	86	9	30	6	13	162
Satellite foci						
Yes	19 (5.06%)	2 (8.69%)	19 (10.55%)	0 (0%)	6 (12%)	51 (5.62%)
No	356 (94.93%)	21 (91.3%)	161 (89.44%)	21 (100%)	44 (88%)	855 (94.37%)
*	98	11	44	7	22	192

**Table 5:** Clinical symptoms of malignancy and histological verification for the Vth region tumours.

Pts N0	Age	Tumour Location	Tumour Diameter	Final Histology	Clinical Symptoms of Malignancy	Radiological Signs of Malignancy
1	54	I_II_III_IV_V	2–4 cm	SCC G3	-	+
2	44	I_II_V	2–4 cm	Low-grade cribriform adenocarcinoma	-	+
3	55	I_II_III_IV_V	>4 cm	SCC G3	FN HB5	+
4	72	I_II_III_IV_V	>4 cm	Adeniod cystic ca, solid type	+	+
5	55	I_II_III_IV_V	>4 cm	Adenoiod cystic ca, solid type	+	+
6	57	II_V	<2 cm	Ca ex PA	-	-
7	47	I_V	<2 cm	Malignant melanoma	-	-
8	59	I_II_V	<2 cm	MALT	-	-
9	94	I_V	2–4 cm	Mucoepidermoid ca, low-grade	-	-
10	50	I_II_V	>4 cm	MALT	-	+
11	42	I_II_V	<2 cm	MALT	-	+
12	49	I_II_III_IV_V	>4 cm	Salivary duct carcinoma	+	+
13	50	I_V	2–4 cm	Epithelial-myoepithelial ca	FN HB3	+
14	75	I_V	2–4 cm	SCC G2	-	-
15	51	II_V	<2 cm	Ca ex PA	-	NA
16	72	I_V	2–4 cm	SCC G2	-	NA
17	28	V	<2 cm	Mucoepidermoid ca	-	-
18	76	V	2–4 cm	Adeniod cystic ca, solid type	-	-
19	48	V	<2 cm	Salivary duct carcinoma	-	-
20	52	V	<2 cm	Salivary duct carcinoma	-	NA
21	39	V	<2 cm	Adeniod cystic ca, solid type	-	NA

NA—no data available; Clinical symptoms of malignancy: + present, - absent; Radiological symptoms of malignancy: + present, - absent; FN HB—facial nerve paresis in House-Brackmann scale; Ca ex Pa—carcinoma ex pleomorphic adenoma; SCC—squamous cell carcinoma; Ca—carcinoma; MALT–oma.

**Table 6:** The logistic regression model in order to establish the relation between predictor and response variables in 28 patients with tumours located exclusively in region V vs. 1901 other patients. The significance threshold at *p* = 0.2.

Predictor Variable (Risk Factor)	Regression Coefficient	Standard Error	Wald χ^2^ Value	*p* Value	OR	95% CI of OR	
						Lower	Upper
Median age	−0.028	2.2343	1.61	0.203	2.979	0.024	2.912
Malignant histology	0.992	1.1214	0.78	0.376	9.719	0.929	1.015
Male gender	−1.917	1.0654	3.23	0.071	2.696	0.136	1.760
Tumour diameter 2–4 cm	−1.792	6.8878	6.8	0.009	2.355	NA	5.806
Clinical symptoms of malignancy (facial nerve paresis)	−16.796	3.4416	2.40	0.996	1.665	0.035	5.911

**Table 7:** The logistic regression model in order to establish relation between predictor variables and response in 100 patients with tumours located exclusively in region V and with extension to neighbouring regions vs. 1829 other patients. The significance threshold at *p* = 0.2.

Predictor Variable (Risk Factor)	Regression Coefficient	Standard Error	Wald χ^2^ Value	*p* Value	OR	95% CI of OR	
						Lower	Upper
Median age	−0.022	0.0097	5.12	0.023	0.978	0.959	0.997
Malignant histology	1.452	0.4533	10.27	0.001	4.275	1.679	10.068
Male gender	−0.292	0.3088	0.89	0.343	0.746	0.401	1.355
Tumour diameter >4 cm	−1.063	0.5524	3.7	0.054	0.345	0.105	0.955
Tumour diameter 2–4 cm	−0.532	0.3246	2.7	0.100	0.587	0.313	1.125
Clinical symptoms of malignancy (facial nerve paresis)	−0.229	0.6223	0.13	0.712	0.795	0.217	2.563

## Data Availability

The datasets used and analysed during the current study are available from the corresponding author on reasonable request.

## References

[1] Frommer J. (1977). The human accessory parotid gland: Its incidence, nature, and significance. Oral Surg. Oral Med. Oral Pathol..

[2] Toh H., Kodama J., Fukuda J., Rittman B., Mackenzie I. (1993). Incidence and histology of human accessory parotid glands. Anat. Rec..

[3] Ahn D., Yeo C.K., Han S.Y., Kim J.K. (2017). The accessory parotid gland and facial process of the parotid gland on computed tomography. PLoS ONE.

[4] Quer M., Guntinas-Lichius O., Marchal F., Poorten V.V., Chevalier D., León X., Eisele D., Dulguerov P. (2016). Classification of parotidectomies: A proposal of the European Salivary Gland Society. Eur. Arch. Oto-Rhino-Laryngol..

[5] Olejniczak I., Leduchowska A., Kozłowski Z., Pietruszewska W. (2021). Evaluation of benign tumors of large salivary glands according to the new classification of the European Salivary Gland Society. Otolaryngol. Pol..

[6] Espin-Ferra J., Merida-Velasco J., Garcia-Garcia J., Sanchez-Montesinos I., Barranco-Zafra R. (1991). Relationships between the parotid gland and the facial nerve during human development. J. Dent. Res..

[7] Guizetti B., Radlanski R. (1996). Development of the parotid gland and its closer neighboring structures in human embryos and fetuses of 19–67 mm CRL. Ann. Anat.—Anat. Anz..

[8] Tamiolakis D., Chimona T.S., Georgiou G., Proimos E., Nikolaidou S., Perogamvrakis G., Papadakis C.E. (2009). Accessory parotid gland carcinoma ex pleomorphic adenoma. Case study diagnosed by fine needle aspiration. Stomatologija.

[9] Ramachar S., Huliyappa H. (2012). Accessory parotid gland tumors. Ann. Maxillofac. Surg..

[10] Afify S.E., Maynard J.D. (1992). Tumours of the accessory lobe of the parotid gland. Heart.

[11] Colella G., Apicella A., Bove P., Rossiello L., Trodella M., Rossiello R. (2010). Oncocytic Carcinoma of the Accessory Lobe of the Parotid Gland. J. Craniofacial Surg..

[12] Rauso R., Colella G., Franco R., Ronchi A., Chirico F. (2019). Ossified Carcinoma Ex Pleomorphic Adenoma in accessory lobe of parotid gland: Complexity in clinical, imaging and histologic diagnosis and minimally invasive surgery. Oral Oncol..

[13] Newberry T.R., Kaufmann C.R., Miller F.R. (2014). Review of accessory parotid gland tumors: Pathologic incidence and surgical management. Am. J. Otolaryngol..

[14] Kakuki T., Takano K., Kurose M., Kondo A., Okuni T., Ogasawara N., Himi T. (2016). Accessory parotid gland tumours: A series of 4 cases. Ear Nose Throat J..

[15] Lin D.T., Coppit G.L., Burkey B.B., Netterville J.L. (2004). Tumors of the accessory lobe of the parotid gland: A 10-year experience. Laryngoscope.

[16] Klotz D.A., Coniglio J.U. (2000). Prudent management of the mid-cheek mass: Revisiting the accessory parotid gland tumor. Laryngoscope.

[17] Choi H.J., Lee Y.M., Kim J.H., Tark M.S., Lee J.H. (2012). Wide excision of accessory parotid gland with anterior approach. J. Craniofacial Surg..

[18] Han X., Zhang X., Gao Y., Pang P., Liu F., Sun C. (2018). Management and prognosis of cancers in the accessory parotid gland. J. Int. Med. Res..

[19] Ma H., Jin S., Du Z., Wang L., Zhang Z., Wang Y. (2018). Pathology and management of masses in the accessory parotid gland region: 24-Year experience at a single institution. J. Cranio-Maxillofac. Surg..

[20] Pasick L.J., Tong J.Y., Benito D.A., Thakkar P., Goodman J.F., Joshi A.S. (2020). Surgical management and outcomes of accessory parotid gland neoplasms: A systematic review. Am. J. Otolaryngol..

[21] Lenzi R., Matteucci J., Muscatello L. (2020). Endoscopic transoral approach to accessory parotid gland. Auris Nasus Larynx.

[22] Mani S., Mathew J., Thomas R., Michael R.C. (2019). Feasibility of Transoral Approach to Accessory Parotid Tumors. Cureus.

[23] Voora R.S., Stramiello J., Funk E., Califano J. (2021). Transoral Excision of a Large Accessory Parotid Gland Tumor. Ear Nose Throat J..

[24] Hasegawa K., Sukegawa S., Ono S., Ando M., Shibata A., Sukegawa-Takahashi Y., Fujimura A., Matsuyama T., Ibaragi S., Nagatsuka H. (2021). Endoscopic-assisted resection of pleomorphic adenoma in the accessory parotid gland. J. Med. Investig..

[25] Xu X., Zhang X., Shi H., Liu W. (2022). Transoral finger-retraction for surgical resection of benign tumors involving masseter muscle and buccal space. J. Dent. Sci..

[26] Hamza M., Anand G. (2023). Resection of Accessory Parotid Gland Tumors: A Multidisciplinary Feat and Review of Literature. Cureus.

[27] Johnson F.E., Spiro R.H. (1979). Tumours arising in accessory parotid tissue. Am. J. Surg..

[28] Perzik S.L., White I.L. (1966). Surgical management of preauricular tumours of the accessory parotid apparatus. Am. J. Surg..

[29] Luksic I., Mamic M., Suton P. (2019). Management of accessory parotid gland tumours: 32-year experience from a single in-stitution and review of the literature. Int. J. Oral Maxillofac. Surg..

[30] Koudounarakis E., Karatzanis A., Nikolaou V., Velegrakis G. (2013). Pleomorphic adenoma of the accessory parotid gland misdiagnosed as glomus tumour. JRSM Short Rep..

[31] Nakatsuka S., Fujiyama H., Takeda K., Kitamura K., Kimura H., Nagano T., Ito M., Asada Y. (2011). An invasive adenocarcinoma of the accessory parotid gland: A rare example developing from a low-grade cribriform cystadenocarcinoma?. Diagn. Pathol..

[32] Gatta G., Guzzo M., Locati L.D., McGurk M., Prott F.J. (2010). Major and minor salivary gland tumours. Crit. Rev. Oncol. Hematol..

[33] Liu C.C., Jethwa A.R., Khariwala S.S., Johnson J., Shin J.J. (2016). Sensitivity, specificity, and posttest probability of parotid fine-needle aspiration: A systematic review and meta-analysis. Otolaryngol. Head Neck Surg..

[34] Piwowarczyk K., Bartkowiak E., Klimza H., Greczka G., Wierzbicka M. (2020). Review and characteristics of 585 salivary gland neoplasms from a tertiary hospital registered in the Polish National Major Salivary Gland Benign Tumors Registry over a period of 5 years: A prospective study. Otolaryngol. Pol..

[35] Kucharska E., Rzepakowska A., Cieślik M., Wilemska S., Bara M., Osuch-Wójcikiewicz E., Niemczyk K. (2022). Indications for surgical treatment of major salivary glands pathologies with epidemiology analysis in adults—Cohort study of 1173 cases. Otolaryngol. Pol..

[36] Wierzbicka M., Fijuth J., Składowski K., Jurkiewicz D., Burduk P., Miłoński J., Niemczyk K., Pietruszewska W., Rogowski M., Stodulski D. (2022). Adjuvant radiotherapy in parotid gland pleomorphic adenoma—Recommendations. Otolaryngol. Pol..

[37] Liu X., Pan Y., Zhang X., Sha Y., Wang S., Li H., Liu J. (2023). A Deep learning Model for Classification of Parotid Neoplasms Based on Multimodal Magnetic Resonance Image Sequences. Laryngoscope.

